# Immunomodulatory Properties of Umbilical Cord Blood-Derived Small Extracellular Vesicles and Their Therapeutic Potential for Inflammatory Skin Disorders

**DOI:** 10.3390/ijms22189797

**Published:** 2021-09-10

**Authors:** Sílvia C. Rodrigues, Renato M. S. Cardoso, Patricia C. Freire, Cláudia F. Gomes, Filipe V. Duarte, Ricardo Pires das Neves, Joana Simões-Correia

**Affiliations:** 1 Exogenus Therapeutics, S.A., 3060-197 Cantanhede, Portugal; silviacouto25@gmail.com (S.C.R.); renatocardoso83@gmail.com (R.M.S.C.); patricia.freire@exogenus-t.com (P.C.F.); claudiafgomes.94@gmail.com (C.F.G.); filipevalenteduarte@gmail.com (F.V.D.); ricardo.neves@uc-biotech.pt (R.P.d.N.); 2 Doctoral Programme in Experimental Biology and Biomedicine (PDBEB), CNC—Center for Neuroscience and Cell Biology, University of Coimbra, 3004-517 Coimbra, Portugal; 3 CNC—Center for Neuroscience and Cell Biology, CIBB—Centre for Innovative Biomedicine and Biotechnology, University of Coimbra, 3004-517 Coimbra, Portugal; 4 IIIUC—Institute of Interdisciplinary Research, University of Coimbra, 3030-789 Coimbra, Portugal

**Keywords:** extracellular vesicles, EV, umbilical cord blood, inflammatory skin disease, psoriasis

## Abstract

Umbilical cord blood (UCB) has long been seen as a rich source of naïve cells with strong regenerative potential, likely mediated by paracrine signals. More recently, small extracellular vesicles (sEV), such as exosomes, have been shown to play essential roles in cell-to-cell communication, via the transport of numerous molecules, including small RNAs. Often explored for their potential as biomarkers, sEV are now known to have regenerative and immunomodulating characteristics, particularly if isolated from stem cell-rich tissues. In this study, we aim to characterize the immunomodulating properties of umbilical cord blood mononuclear cell-derived sEV (UCB-MNC-sEV) and explore their therapeutic potential for inflammatory skin diseases. UCB-MNC-sEV were shown to shift macrophages toward an anti-inflammatory phenotype, which in turn exert paracrine effects on fibroblasts, despite previous inflammatory stimuli. Additionally, the incubation of PBMC with UCB-MNC-sEV resulted in a reduction of total CD4^+^ and CD8^+^ T-cell proliferation and cytokine release, while specifically supporting the development of regulatory T-cells (Treg), by influencing *FOXP3* expression. In a 3D model of psoriatic skin, UCB-MNC-sEV reduced the expression of inflammatory and psoriatic markers *IL6*, *IL8*, *CXCL10*, *COX2*, *S100A7*, and *DEFB4*. In vivo, UCB-MNC-sEV significantly prevented or reversed acanthosis in imiquimod-induced psoriasis, and tendentially increased the number of Treg in skin, without having an overall impact on disease burden. This work provides evidence for the anti-inflammatory and tolerogenic effect of UCB-MNC-sEV, which may be harnessed for the treatment of Th17-driven inflammatory skin diseases, such as psoriasis.

## 1. Introduction

Virtually every living cell releases extracellular vesicles (EV), which can be classified based on size and marker expression. One of the smallest known groups of EV, exosomes, have a diameter typically ranging from 30 to 100 nm and originate from endosomes [1]. Composed of lipids, proteins and nucleic acids, these ubiquitous vesicles are thought to be involved in multiple diseases, including inflammatory and autoimmune skin conditions [2]. Due to their physical characteristics, which allow them to carry molecules across long distances, EV are often explored for their potential as biomarkers [3,4]. Physiologically, small EV (sEV), such as exosomes, are key mediators of cellular communication, namely through microRNAs [5]. Hence, depending on the producing cell, sEV may have modulating characteristics, which can potentially be harnessed for therapeutic purposes. In fact, these naturally-produced vesicles are currently explored for the treatment of several conditions, including wound healing [6] and autoimmune diseases [7,8,9]. Their use replaces cell therapies [10,11], while conferring advantages, namely concerning handling and formulation.

Chronic inflammatory skin disorders, such as psoriasis, represent a significant medical, psychological and financial burden to patients and healthcare systems [12,13]. Plaque psoriasis is characterized by patches of red, dry, and scaly skin, which often appear on the elbows, knees, and scalp. IL-17A and its upstream regulator IL-23 are two key molecules in the pathogenesis of psoriasis, and approaches that specifically target this pathway showed great clinical responses [14]. Despite these recent encouraging advances, continuing innovation is key for the development of new therapeutic approaches for patients who still have unmet needs, namely an incomplete treatment response, contraindications, or even affordability and practicality [15].

Umbilical cord blood (UCB) is a rich source of stem cells and immature T-cells [16] with potent suppressive ability [17]. The collection of UCB, commonly seen as medical waste, is non-invasive and has limited ethical concerns. We have previously shown that sEV from UCB mononuclear cells (UCB-MNC-sEV), produced using an established optimized protocol [18], accelerate the healing of diabetic wounds [6] and have a good safety profile [19]. In this study, we characterize the immunomodulating properties of UCB-MNC-sEV, and explore their potential in the treatment of psoriasis symptoms.

## 2. Results

### 2.1. UCB-MNC-sEV Have Anti-Inflammatory and Tolerogenic Effects, Modulating Different Immune Players Directly and Indirectly

While exploring the therapeutic potential of UCB-MNC-sEV for chronic wound healing [6,18], we found differences in the immunological profile of treated versus control skin. Namely, 194 genes associated with immune system processes or inflammatory responses, corresponding to about 16% of all measured genes, were differentially expressed between the two groups of animals (Figure S1). Based on these results and on existing literature, we performed a series of in vitro proof-of-concept experiments to determine the nature and strength of UCB-MNC-sEV’s immunomodulatory effect.

**Figure 1:**
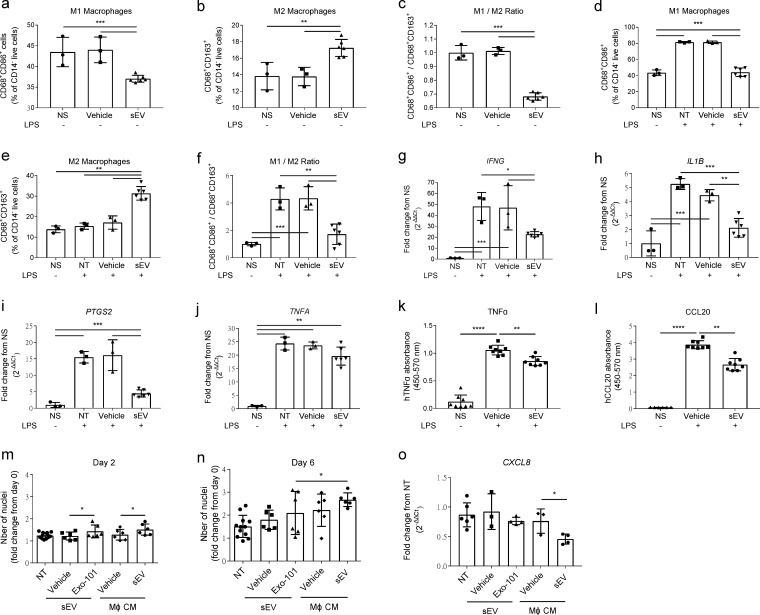
UCB-MNC-sEV’s immunomodulatory effects in vitro. (**a**–**f**) THP-1-derived macrophages, with or without LPS stimulation as indicated, were incubated with 1 × 10^10^ particles/mL of UCB-MNC-sEV for 24 h, before flow cytometry analysis (*n* ≥ 3). (**g**–**j**) Relative expression of M1-associated genes by qPCR (*n* ≥ 3) and (**k**,**l**) release of pro-inflammatory molecules by ELISA (*n* ≥ 6). (**m**,**n**) Proliferation of normal human dermal fibroblasts, 2 and 6 days after incubation with UCB-MNC-sEV (1 × 10^10^ particles/mL) or media from UCB-MNC-sEV-stimulated macrophages (*n* ≥ 6), and (**o**) CXCL8 expression at 48h (*n* ≥ 3). All results are presented as mean ° SD. * *p* ≤ 0.05, ** *p* ≤ 0.01, *** *p* ≤ 0.001, **** *p* ≤ 0.0001. NS, non-stimulated; NT, non-treated; CM, conditioned media.

In line with studies using sEV from different sources [20,21], UCB-MNC-sEV promote the differentiation of macrophages into an anti-inflammatory M2, rather than a pro-inflammatory M1 phenotype (Figure 1). This effect is present both in unstimulated (Figure 1a–c) and LPS-stimulated macrophages (Figure 1d–f) and correlates with the de novo synthesis and release of inflammatory mediators (Figure 1a–l). Specifically, UCB-MNC-sEV administration significantly decreases the level of *IFNG*, *IL1B*, *PTSG2,* and *TNFA* mRNA in macrophages, as well as the release of TNFα and CCL20 proteins. These data strongly indicate that UCB-MNC-sEV play a direct role in macrophage regulation, having an anti-inflammatory effect, even in the context of an acute pro-inflammatory stimulus.

At the local level, UCB-MNC-sEV directly promote the proliferation of dermal fibroblasts (Figure 1m,n). Interestingly, the incubation of fibroblasts with conditioned medium from UCB-MNC-sEV-stimulated macrophages likewise modulates cell proliferation, as well as chemokine synthesis, with a more pronounced effect when compared to direct UCB-MNC-sEV stimulation (Figure 1o). These results suggest that, while UCB-MNC-sEV do act directly on target skin cells, their effects are likely reinforced by indirect mechanisms, depending on neighboring immune cell populations, such as macrophages.

**Figure 2:**
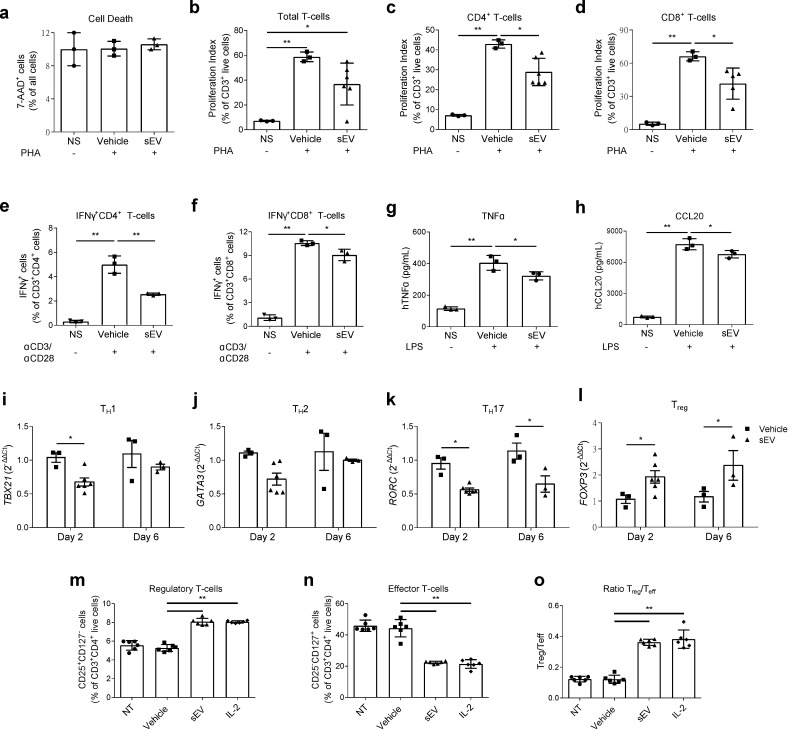
UCB-MNC-sEV’s immunomodulatory effects on T-cells in vitro**.** (**a**–**f**) Human PBMC, stimulated with PMA or αCD3/αCD28 as indicated, were incubated with a single dose of UCB-MNC-sEV at 1 × 10^10^ particles/mL for 6 days, followed by flow cytometry analysis (*n* ≥ 3). (**g**,**h**) TNFα and CCL20 release by LPS-stimulated PBMC after a 24 h incubation with 1 × 10^10^ particles/mL UCB-MNC-sEV (*n* = 3). (**i**–**l**) Relative expression of transcription factors by PBMC incubated with UCB-MNC-sEV for 2 or 6 days (*n* ≥ 3). Cells were sorted based on FSC, SSC, CD3, and CD4, prior to RNA extraction. (**m–o**) Phenotyping of αCD3/αCD28-activated PBMC, after 6 days of incubation with UCB-MNC-sEV at 1 × 10^10^ particles/mL or IL-2 (100 IU/mL) and TGF-β (5 ng/mL) (*n* = 6). All results are presented as mean ° SD. * *p* ≤ 0.05, ** *p* ≤ 0.01. NS, non-stimulated; NT, non-treated.

To evaluate the response of T-cells to UCB-MNC-sEV stimulation, in a physiological context, whole human PBMC were incubated for 6 days with a single dose of 1 × 10^10^ vesicles/mL, following activation with α-CD3/-CD28. UCB-MNC-sEV significantly reduced the proliferation of total, CD4^+^ and CD8^+^ T-cells, as well as the intracellular content of IFNγ in each of these populations (Figure 2a–f). The release of TNFα and CCL20 by total PBMCs was also significantly decreased (Figure 2g,h). Notably, after two days of treatment, UCB-MNC-sEV modulated the expression of population-specific transcription factors in CD4^+^ T-cells, with a trend toward the reduction of GATA-3, a significant decrease in T-bet and RORγt, and a significant increase in Foxp3 mRNA (Figure 2i–l). This effect was still visible after 6 days of treatment, for RORγt and Foxp3 expression.

To validate the differences in transcriptional regulation, we determined the populational T-cell changes by flow cytometry. As expected, UCB-MNC-sEV treatment promoted the differentiation into regulatory T-cells (Treg), while inhibiting effector populations (Figure 2m–o). In this experiment, Tregs were defined as CD4^+^CD25^+^CD127^-^ cells, and the results were confirmed using a gating strategy based on the expression of the transcription factor Foxp3 (Figure S2). Importantly, UCB-MNC-sEV’s effect was shown to be as powerful as IL-2 for the induction of Treg (Figure 2m and Figure S2).

### 2.2. UCB-MNC-sEV Reduce the Expression of Psoriasis Markers, in an In Vitro 3D Model

**Figure 3:**
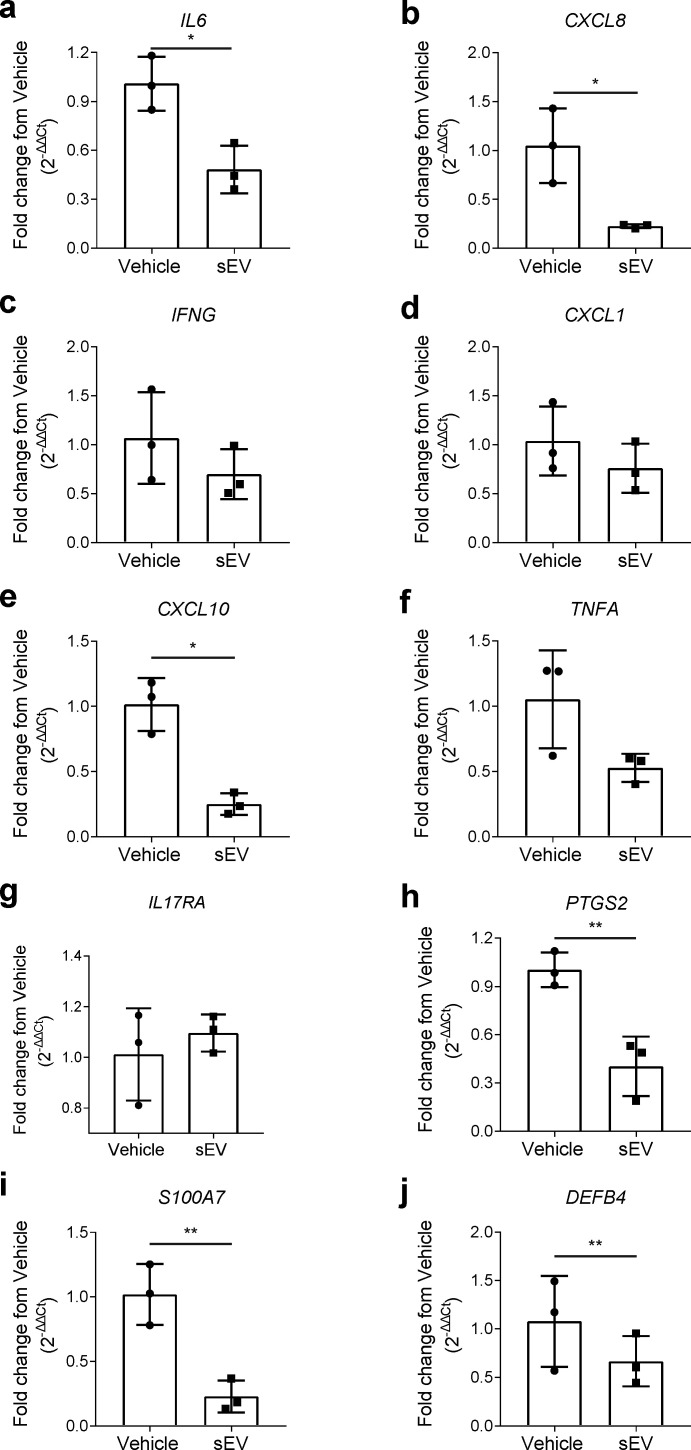
Decreased expression of epidermal psoriatic features after UCB-MNC-sEV treatment. Relative expression of (**a**–**h**) pro-inflammatory mediators and (**i**,**j**) anti-microbial peptides by a 3D model of human psoriatic epidermis, treated with 1 × 10^10^ particles/mL UCB-MNC-sEV twice daily for 6 days (*n* = 3). All results are presented as mean ° SD. * *p* ≤ 0.05, ** *p* ≤ 0.01.

Considering the results from Figure 2, which evidenced an immunomodulatory effect of UCB-MNC-sEV, particularly focused on the Th17 and Treg response, we decided to evaluate the therapeutic benefit of UCB-MNC-sEV in psoriasis. Using an in vitro 3D model of reconstructed human epidermis, engineered to be histologically and metabolically similar to psoriatic skin, we show that UCB-MNC-sEV treatment significantly reduced the expression of the inflammatory mediators IL-6, IL-8, CXCL10, and COX-2, and had a tendential effect on *IFNG* and *TNFA* mRNA (Figure 3a–h). Moreover, the expression of psoriasin (S100A7) and beta-defensin-2 (DEFB4), two psoriasis-associated antimicrobial peptides, was also significantly decreased (Figure 3i,j).

### 2.3. UCB-MNC-sEV Show a Modest Effect in Imiquimod-Induced Psoriasis, Regulating Keratinocyte Proliferation and T-Cell Homeostasis

**Figure 4:**
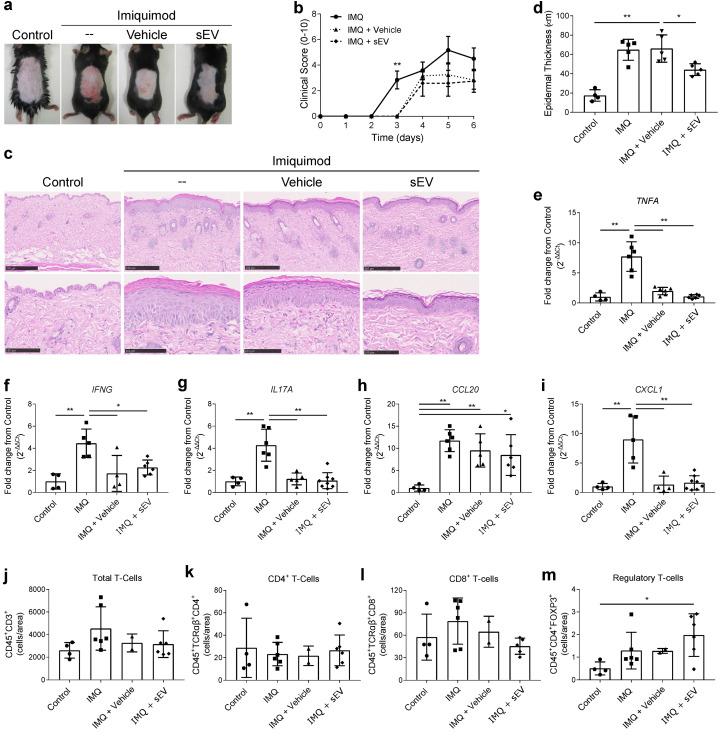
Therapeutic potential of UCB-MNC-sEV for imiquimod-induced psoriasis. C57BL/6 mice received 5% imiquimod (IMQ) on their shaved backs (approx. 7 cm^2^), daily for 6 days. A topical formulation containing 2 × 10^10^ particles/mL UCB-MNC-sEV was applied to the same area every day, 1 h after IMQ. (**a**) Representative photos and (**b**) clinical score (*n* = 6). (**c**) Representative H&E microphotographs used to measure (**d**) epidermal thickness (*n* ≥ 4). Scale bars = 250 µm or 100 µm, respectively, for upper and lower microphotographs. Epidermal thickness was measured from *stratum basale* to *stratum granulosum*, averaging 5 measurements per section, for a total of 20 data points per animal. (**e**–**i**) Relative expression of pro-inflammatory mediators in the skin of all tested groups (*n* 4). (**j**–**m**) Digested skin of all four test groups was analyzed by flow cytometry for identification of total, CD4+, CD8+ and regulatory T-cells (*n* ≥ 2). All results are presented as mean ° SD. * *p* ≤ 0.05, ** *p* ≤ 0.01.

Application of imiquimod, a TLR7 agonist, to mouse skin leads to the development of psoriatic features, such as epidermal thickening, erythema, inflammatory cell infiltration, and epidermal expression of IL-17 [22]. Daily topical treatment with UCB-MNC-sEV, dissolved in a slow-release hydrogel and applied 1 h after imiquimod, did not significantly ameliorate macroscopic psoriasis-like features, when compared to hydrogel alone (Figure 4a,b). However, UCB-MNC-sEV were significantly superior to hydrogel at reducing acanthosis, as seen by microphotographs (Figure 4c,d). The expression of the inflammatory markers TNFα, IFNγ, IL-17A, CCL20, and CXCL1, albeit significantly reduced when compared to the imiquimod control, was similar between the two groups receiving hydrogel, with or without UCB-MNC-sEV (Figure 4e–i). When comparing the three treatment groups, there were no major changes in the skin infiltration of most inflammatory cells, including neutrophils, macrophages, total T-cells, γδ T-cells, and total CD4^+^ T-cells (Figure 4j,k and Figure S3). CD8^+^ T-cells were slightly reduced in UCB-MNC-sEV-treated animals compared to the skin of mice receiving only hydrogel (Figure 4l). Furthermore, UCB-MNC-sEV tendentially increased the number of Treg in the skin of imiquimod-treated mice (Figure 4m), a finding that is consistent with gene expression data from diabetic mice with chronic skin wounds (Figure S4) [6,18]. These findings indicate that, in a context of psoriasis, UCB-MNC-sEV improve certain pathological features, possibly through a mechanism that involves both local and immune cells, but did not significantly reduce the overall disease burden in this model.

## 3. Discussion

Over the last years, sEV have been explored for their potential as cell-free immunomodulatory and regenerative agents. Indeed, unmodified or engineered sEV were shown to have therapeutic potential across multiple conditions, including cancer [23], inflammatory lung diseases [24,25], and autoimmunity [26]. Here, we explore the mechanism of action of sEV isolated from UCB-MNC, and evaluate their effect in psoriasis models.

In vitro, UCB-MNC-sEV exhibit anti-inflammatory properties, affecting macrophage differentiation and cytokine production. These results are consistent with previous findings using sEV isolated from bone marrow [20] or cord blood [21]. We also show that the presence of UCB-MNC-sEV-induced M2 macrophages has a down-stream effect on neighboring cells, such as skin fibroblasts, reducing their response to an inflammatory trigger. Moreover, UCB-MNC-sEV strongly inhibited cell proliferation and cytokine production by LPS-stimulated total CD4^+^ and CD8^+^ T-cells, consistent with previous reports [11,27]. This outcome is possibly due to an effect on T-cell differentiation, given that UCB-MNC-sEV promote a shift from a Th1 or Th17 into a Treg phenotype. Notably, UCB-MNC-sEV stimulus was shown to be as effective as IL-2 in promoting Treg development.

Our in vitro findings evidenced a potential mechanism of action for UCB-MNC-sEV, responsible for a shift in the expression of transcription factors, which favor Treg differentiation and concomitantly silence Th17 signaling. Biologics targeting the Th17 axis (α-IL-17 and α-IL-23) have been proved to be clinically effective in ameliorating psoriasis symptoms [28,29,30]. Additionally, previous reports suggest that Treg, a typically tolerogenic cell population, play a crucial role in the maintenance of skin homeostasis. Treg-deficient animals present an exacerbated response to imiquimod [31] and Treg from psoriatic patients display an impaired suppressive function [32]. Hence, we hypothesized that UCB-MNC-sEV’s profile could be therapeutically beneficial in psoriasis. To test this, we employed a model of reconstructed human epidermis, composed of keratinocytes in various stages of differentiation, and pre-treated to display psoriasis-like inflammatory features. UCB-MNC-sEV treatment significantly reduced the expression of psoriasis-associated molecules, including IL-6 and IL-8, as well as antimicrobial peptides S100A7 and DEFB4, thereby supporting its therapeutic potential for this disease.

In order to test UCB-MNC-sEV’s effect in vivo, we first designed a micelle-rich hydrogel that solidifies at normal body temperature, thus reducing product loss when applied to the skin. Psoriasis-like symptoms were induced by topical applications of imiquimod, and hydrogel was applied 1 h later, alone or containing UCB-MNC-sEV. In this in vivo model, UCB-MNC-sEV proved superior to hydrogel in reducing or preventing keratinocyte hyperproliferation, as measured by epidermal thickness. Yet, there were no significant differences in the disease scores, mRNA expression and cellular profile between the two treatment groups. While it is possible that the application of hydrogel alone strongly improves psoriatic symptoms, the results observed are better explained by the possible trapping of imiquimod molecules in hydrogel micelles, therefore preventing full symptom development. An alternative experimental setting would either allow for a longer time interval between imiquimod and test treatment applications and/or require the increase of imiquimod dosage. Nevertheless, in line with previous in vitro data, UCB-MNC-sEV had a positive effect on keratinocytes and were tendentially stronger than hydrogel alone in shifting skin cellular infiltrates toward a tolerogenic profile. Importantly, gene expression data from chronic wounds support the increase in Treg differentiation following UCB-MNC-sEV treatment. Given the incomplete therapeutic response of imiquimod-treated animals to UCB-MNC-sEV, it is plausible that UCB-MNC-sEV could act as an adjuvant treatment, in combination with standard therapies, such as anti-IL-17A or anti-IL23. This strategy would not only target immune-driven disease pathways, but also simultaneously stimulate repair mechanisms in the skin.

**Figure 5:**
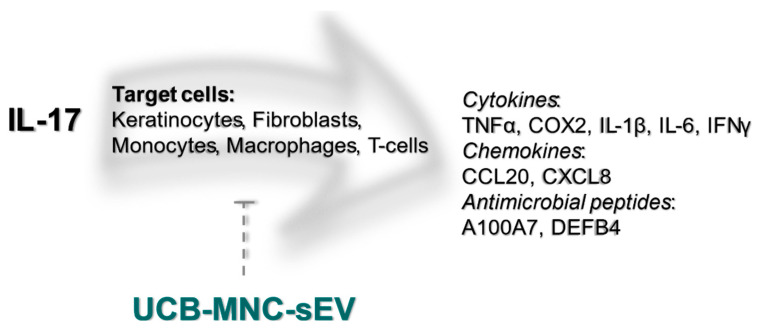
UCB-MNC-sEV’s putative effect on the IL-17 signaling pathway. IL-17, particularly IL-17A, is thought to be a crucial driver of psoriatic disease. IL-17 target cells include keratinocytes, fibroblasts, monocytes, macrophages, and T-cells, all of which are likewise targeted by UCB-MNC-sEV, either directly or indirectly. As shown in this paper, treatment with UBC-MNC-sEV affects multiple players of the IL-17 cascade, and results in a lower expression and/or release of psoriasis-associated mediators, such as TNFα, COX2, IL-1β, IL-6, IFNγ, CCL20, CXCL8, A100A7 and DEFB4.

In conclusion, we show that UCB-MNC-sEV decrease inflammation by targeting different cell populations, such keratinocytes, fibroblasts, and macrophages, and by modulating T-cell differentiation and cytokine production (Figure 5). These findings warrant further proof-of-concept studies on the therapeutic potential of UCB-MNC-sEV in inflammatory skin conditions, in particular diseases thought to benefit from Th17/Treg-targeting.

## 4. Materials and Methods

### 4.1. UCB Collection, Testing, and Data Protection

Human UCB samples and relevant donor information were collected following signed informed consent, under approval of the Portuguese National Data Protection Committee and the ethics committees from five Portuguese hospitals, according to local legislation and following the principles of the Declaration of Helsinki. UCB processing, including microbiological testing, and storage were performed by an accredited biobank (Stemlab, S.A, Cantanhede, Portugal).

### 4.2. Cell Culture

#### 4.2.1. UCB-MNC

Isolated UCB-MNC were cultured under 0.5% O_2_, for 18 h, at a density of 2 million cells/mL in serum-free cell culture medium (Lonza AG, Basel, Switzerland) supplemented with 0.5 μg/mL FMS-like tyrosine kinase-3 (Peprotech, London, UK) and 0.5 μg/mL stem-cell factor (Peprotech, London, UK).

#### 4.2.2. THP-1-Derived Macrophages

THP-1 cells (ATCC, Manassas, VA, USA) were grown for 72 h in the presence 25 nM PMA (Sigma, St. Louis, MO, USA). THP-1-derived macrophages were then stimulated for 24 h with 1 μg/mL LPS (Sigma, St. Louis, MO, USA), followed by a 24-h incubation with 1 × 10^10^ particles/mL UCB-MNC-sEV, when indicated.

#### 4.2.3. Dermal Fibroblasts

Normal human dermal fibroblasts were kept in Fibroblast Basal Medium, supplemented with Fibroblast Growth Kit Serum-Free, Phenol Red and Penicillin-Streptomycin-Amphotericin B Solution (ATCC, Manassas, VA, USA). When appropriate, cells were counted after Hoescht staining (VWR International, Radnor, PA, USA).

#### 4.2.4. PBMC

Fresh human PBMC samples were isolated from volunteer donors, following informed consent, by density gradient centrifugation (Stemcell Technologies, Vancouver, Canada). For T-cell experiments, 2 × 10^5^ cells/well were activated with PMA (Sigma, St. Louis, MO, USA), anti-CD3/-CD28 (see Table 1 or LPS (Sigma, St. Louis, MO, USA), as indicated, followed by a single dose of UCB-MNC-sEV at 1 × 10^10^ particles/mL.

### 4.3. UCB-MNC-sEV Isolation

UCB-MNC culture media were subjected to an optimized isolation process, combining ultrafiltration and size exclusion chromatography [18]. The resulting vesicles were characterized by transmission electron microscopy, flow cytometry, mass spectrometry for protein and lipid composition, RNA sequencing, and nanoparticle tracking analysis, and were found to be rich in CD63 and smaller than 200 nm [18].

### 4.4. Gene Expression

Total RNA was extracted from cells (THP-1, NHDF, human PBMC, human 3D skin) and tissue (murine skin) with RNeasy Mini Kit (Qiagen, Hilden, Germany) and analyzed with total RNA chips in Bioanalyzer 2100 (Agilent Technologies, Santa Clara, CA, USA). SuperScript™ IV VILO™ Master Mix (ThermoFisher Scientific, Waltham, MA, USA) was used for reverse transcription and gene expression was detected by qPCR, using the primer pairs in Table 2.

### 4.5. Protein Quantification and Flow Cytometry (Human)

TNFα and CCL20 were measured by ELISA (BioLegend, San Diego, CA, USA). M1 macrophages were defined as CD14^-^CD68^+^CD86^+^, and M2 macrophages as CD14^-^CD68^+^CD163^+^ by flow cytometry. Tregs were defined as CD4^+^CD25^+^CD127^-^ cells and the results were confirmed using a gating strategy based on the expression of the transcription factor *FOXP3* (Figure S2). Foxp3 expression was analyzed by flow cytometry using the Foxp3/Transcription Factor Staining Buffer Set (eBioscience, San Diego, CA, USA). Whenever mentioned, IL-2 was used as a positive control for Treg development. Antibody details can be found in Table 2.

### 4.6. Reconstructed Psoriatic Human Epidermis

The 3D model of “psoriasis-like” human epidermis (Sterlab, Vallauris, France) was used according to the manufacturer’s instructions. Briefly, epidermal inserts were placed in a 12-well feeder-plate and allowed to equilibrate in the supplied culture medium for one day at 37 °C and 5% CO_2_, before treatment with 1 × 10^10^ particles/mL UCB-MNC-sEV, twice a day for 5 consecutive days.

### 4.7. UCB-MNC-sEV Formulation for In Vivo Topical Use

UCB-MNC-sEV are typically formulated in a saline solution. For in vivo experiments, UCB-MNC-sEV were dissolved in a micellar hydrogel, which solidifies at body temperature, thereby reducing product loss when applied to the skin and allowing for a slow particle release (not shown).

### 4.8. Imiquimod-Induced Psoriasis

Animal experiments were approved by the ethical committee of the Spanish National Cardiovascular Research Center, and performed according to national and European regulations, respecting animal welfare guidelines and the 3R’s rule. Eight- to twelve-week-old C57BL/6 mice received daily applications of imiquimod (3M Pharmaceuticals, Saint Paul, MN, USA) on their shaved backs, for 6 consecutive days. One hour after every imiquimod application, 3 × 10^9^ particles/cm^2^ UCB-MNC-sEV dissolved in hydrogel were delivered topically. Epidermal thickness was measured on H&E-stained samples, using the software NanoZoomer Digital Pathology NDP.view2 (Hamamatsu Photonics, Hamamatsu, Japan), from stratum basale to stratum granulosum, averaging 5 measurements per section, for a total of 20 data points per animal. For flow cytometry, skin was digested with 0.25 mg/mL Liberase (Roche, Basel, Switzerland) in serum-free RPMI, for 60 min at 37 °C. Skin cell suspensions were stained with fluorescently labelled antibodies, following Fc Block (Table 2). Absolute cell counts were performed using Trucount tubes (BD Biosciences, San Jose, CA, USA). RNA analyses were performed as described above.

### 4.9. Statistical Analyses

Data analyses were performed with Prism 6 (GraphPad Software, San Diego, CA, USA). Unpaired t-tests or one-way ANOVA were employed whenever appropriate (*p* < 0.05).

## Figures and Tables

**Table 1:** Human and mouse antibodies for flow cytometry.

Target	Clone	Manufacturer
**Anti-human antibodies**		
CD14	M5E2	BD Biosciences, San Jose, CA, USA
CD163	GHI/61	BD Biosciences, San Jose, CA, USA
CD28	CD28.2	BioLegend, San Diego, CA, USA
CD3	OKT3	BioLegend, San Diego, CA, USA
CD68	Y1/82A	BD Biosciences, San Jose, CA, USA
CD86	FUN-1	BD Biosciences, San Jose, CA, USA
IL-2	MQ1-17H12	BioLegend, San Diego, CA, USA
**Anti-mouse antibodies**		
CD11b	M1/70	BD Biosciences, San Jose, CA, USA
CD11c	HL3	BD Biosciences, San Jose, CA, USA
CD16/CD32	2.4G2	Tonbo Biosciences, San Diego, CA, USA
CD25	PC61.5	eBioscience, San Diego, CA, USA
CD3e	145-2C11	BD Biosciences, San Jose, CA, USA
CD4	RM4-5	BD Biosciences, San Jose, CA, USA
CD45.2	104	eBioscience, San Diego, CA, USA
CD62L	MEL-14	BD Biosciences, San Jose, CA, USA
CD8	53-6.7	BD Biosciences, San Jose, CA, USA
Ly6C	AL-21	BD Biosciences, San Jose, CA, USA
Ly6G	1A8	BD Biosciences, San Jose, CA, USA
TCR γ/δ	GL3	BioLegend, San Diego, CA, USA
TCR α/β	GL2	BioLegend, San Diego, CA, USA

**Table 2:** Human and mouse primer sequences for qPCR.

Target Gene	Forward Sequence	Reverse Sequence
**Human**		
*CXCL1*	AGGGAATTCACCCCAAGAAC	ACTATGGGGGATGCAGGATT
*CXCL10*	TTCAAGGAGTACCTCTCTCTAG	CTGGATTCAGACATCTCTTCTC
*DEFB4*	ATCAGCCATGAGGGTCTTGT	GAGACCACAGGTGCCAATTT
*FOXP3*	GCTTCATCTGTGGCATCATC	TGGAGGAACTCTGGGAATGT
*GATA3*	CGCCTGCGGGCTCTATC	CCTTCGCTTGGGCTTAATGA
*IFNG*	GGTAACTGACTTGAATGTCC	TTTTCGCTTCCCTGTTTTAG
*IL1B*	CTAAACAGATGAAGTGCTCC	GGTCATTCTCCTGGAAGG
*IL6*	GGTACATCCTCGACGGCATCT	GT GCCTCTTTGCTGCTTTCAC
*IL8*	GTTTTTGAAGAGGGCTGAG	TTTGCTTGAAGTTTCACTGG
*PTGS2*	ATCTACCCTCCTCAAGTCCC	TACCAGAAGGGCAGGATACAG
*RORC*	TGGACCACCCCCTGCTGAGAA	CTTCAATTTGTGTTCTCATGACT
*S100A7*	CCAAACACACACATCTCACTCA	TCAGCTTGAGTGTTGCTCATC
*TBX21*	GATGTTTGTGGACGTGGTCTTG	CTTTCCACACTGCACCCACTT
*TNFA*	AGGCAGTCAGATCATCTTC	TTATCTCTCAGCTCCACG
**Mouse**		
*CCL20*	ACTGTTGCCTCTCGTACATACA	GAGGAGGTTCACAGCCCTTTT
*CXCL1*	GCTTGAAGGTGTTGCCCTCAG	AGAAGCCAGCGTTCACCAGAC
*FOXP3*	CACCCAGGAAAGACAGCAACC	GCAAGAGCTCTTGTCCATTGA
*IFNG*	CGGCACAGTCATTGAAAGCCTA	GTTGCTGATGGCCTGATTGTC
*IL17A*	TTTAACTCCCTTGGCGCAAAA	CTTTCCCTCCGCATTGACAC
*TNFA*	GTTCTATGGCCCAGACCCTCAC	GGCACCACTAGTTGGTTGTCTTTG

## Data Availability

For any data or certificate requests, please contact Exogenus Therapeutics, S.A., at team@exogenus-t.com.

## Supplementary Materials

The following are available online at https://www.mdpi.com/article/10.3390/ijms22189797/s1.
