# Supplementary material for: Immunomodulatory Properties of Umbilical Cord Blood-Derived Small Extracellular Vesicles and Their Therapeutic Potential for Inflammatory Skin Disorders
Source: Int J Mol Sci. 2021 Sep 10;22(18):9797. doi: 10.3390/ijms22189797 (PMC8468428; doi:10.3390/ijms22189797)
Supplement: Supplementary file 1 [file ijms-22-09797-s001.zip › ijms-1351988-supplementary.pdf]

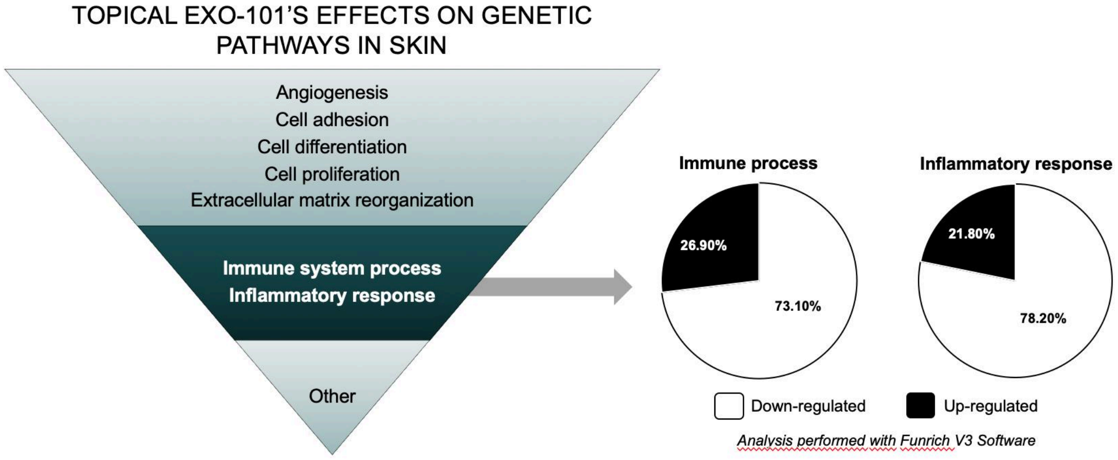


**Figure S1: Effects of Exo-101 treatment on gene expression of chronic skin wounds.** STZ-induced diabetic mice were treated topically with $2.5x10^9$ particles/mL Exo-101, twice per day for 15 days, after which skin mRNA was extracted. The inverted triangle on the left represents the different major processes affected by Exo-101 treatment. The circles on the right illustrate the percentage of inflammation- and immune system-related genes up- or down-regulated after Exo-101 treatment.

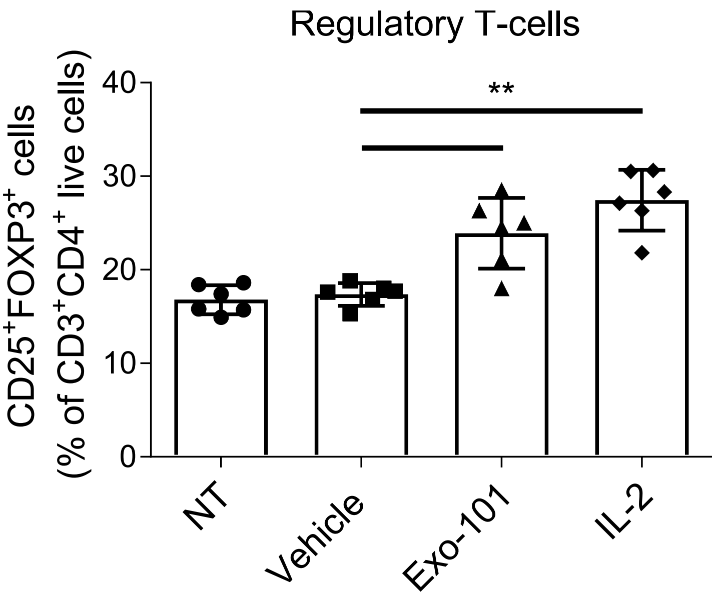


**Figure S2: Effects of Exo-101 treatment on the percentage of regulatory T-cells (Treg).** Human PBMC were activated with αCD3/αCD28 and treated for 6 days with Exo-101 ($1x10^{10}$ particles/mL) or IL-2 (100 IU/ml) and TGF-β (5ng/mL) (n=6). Treg were defined as $CD3^+CD4^+CD25^+FOXP3^+$ cells. Comparison with Figure 1ab, where Treg were identified based on the expression markers CD25 and CD127. All results are presented as mean +/- SD. **p≤0.01. NT, non-treated.

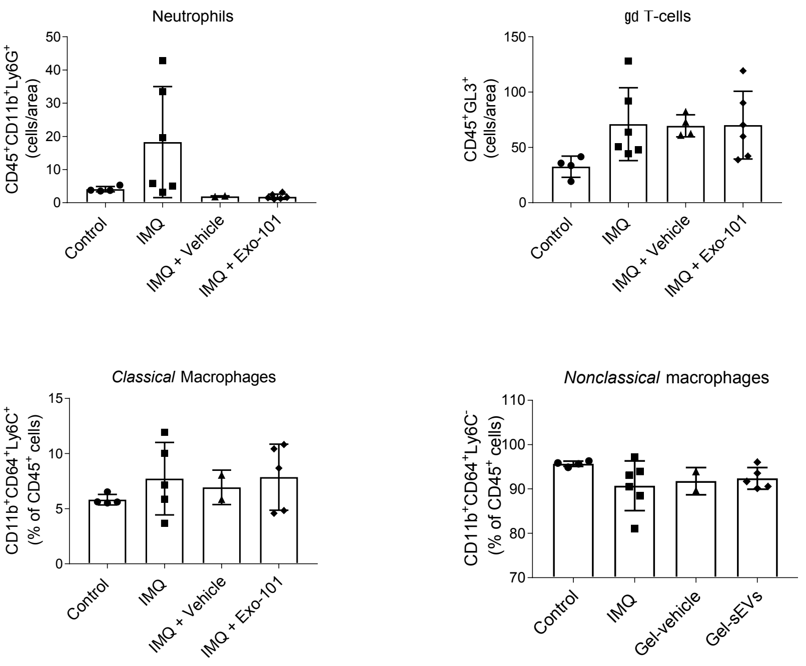


**Figure S3: Cell populations in the skin of imiquimod- and Exo-101 treated animals.** Flow cytometry identification of neutrophils, γδ T-cells and macrophages in digested skin of mice treated with imiquimod (IMQ) and/or Exo-101 as indicated (n≥2). All results are presented as mean +/- SD.

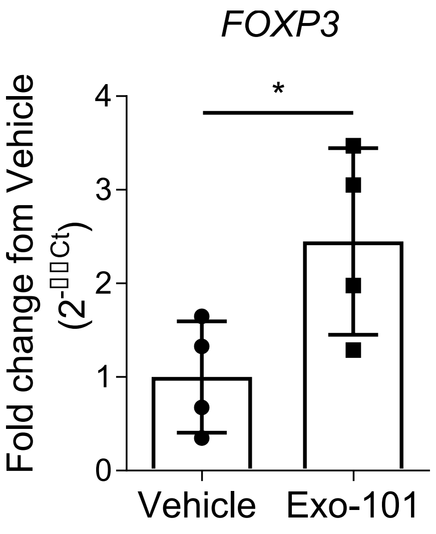


**Figure S4: FOXP3 expression in the skin of Exo-101 treated mice with chronic wounds.** STZ-induced diabetic mice (n=4) were treated topically with $2.5x10^9$ particles/mL Exo-101 or vehicle, twice per day for 15 days, after which skin mRNA was extracted. All results are presented as mean +/- SD. *$p \leq 0.05$.
